# Attention-Enhanced Pedestrian Trajectory Prediction via Compressed Point Cloud Representation

**DOI:** 10.3390/jimaging12070305

**Published:** 2026-07-07

**Authors:** Yuting Han, Shuyu Li, Yunfei Tan

**Affiliations:** School of Artificial Intelligence and Computer Science, Shaanxi Normal University, Xi’an 710119, China; hyt1624@snnu.edu.cn (Y.H.); wkt@snnu.edu.cn (Y.T.)

**Keywords:** pedestrian trajectory prediction, point cloud compression, attention mechanism, multi-feature extraction

## Abstract

To address the high storage overhead and inadequate spatial geometric representation associated with raw point cloud data in multi-pedestrian trajectory prediction, a compressed point cloud-based and attention-enhanced trajectory prediction method (CPCAE) is proposed in the paper. First, for input raw point cloud, a lossy compression module is designed, which improves the Depoco framework by introducing a multi-feature extraction component and employing a coordinate decomposition strategy to optimize compression quality and spatial representation. For input video frames of pedestrians, spatial features are extracted using a 2D convolutional network, and dynamic interactions among pedestrians are captured by a Transformer-based encoder. Then, both spatial attention and modal attention mechanisms are incorporated to dynamically balance the contributions of two modal features and precisely identify key regions and positions. Experimental results evaluate the proposed framework from the perspectives of point cloud compression and downstream trajectory prediction. The results demonstrate that compressed point cloud representations can support competitive trajectory prediction performance in CPCAE.

## 1. Introduction

Pedestrian trajectory prediction plays a crucial role in autonomous driving [1], intelligent surveillance, and intelligent transportation systems [2]. Traditional approaches primarily rely on video frames or historical trajectory data for modeling. However, single-modal information often fails to comprehensively capture the complex and dynamic characteristics of pedestrian motion, thereby constraining prediction accuracy and robustness [3]. In recent years, multimodal trajectory fusion methods have shown substantial promise in improving prediction performance by leveraging complementary information from multiple data sources [4]. Among these, point cloud data offers highly accurate three-dimensional spatial representations [5]. Unlike video data, point clouds contain only positional and geometric features of pedestrians, avoiding sensitive appearance information. This not only enhances spatial modeling capability but also provides stronger privacy protection for trajectory prediction [6].

In terms of model architecture, the encoder–decoder framework has been widely adopted for pedestrian trajectory prediction, as it effectively models the spatiotemporal dynamics of pedestrian movements [7]. The encoder extracts historical trajectory data, point cloud features, and scene contextual cues, encoding them into a latent representation that captures long-range dependencies and interactions between pedestrians and their surroundings [8]. The decoder subsequently predicts future trajectories from this latent space, incorporating adaptive mechanisms to improve both prediction accuracy and behavioral plausibility [9].

However, the high-dimensional sparsity and redundancy of point cloud data continue to pose major challenges for pedestrian trajectory prediction. Therefore, developing an encoder–decoder architecture that reduces point cloud data volume while preserving spatial representations and incorporating historical trajectory information for accurate prediction remains a critical open challenge. To address these challenges, this paper introduces the CPCAE framework, whose main contributions are summarized as follows:

A pedestrian trajectory prediction model based on an encoder–decoder is proposed, which leverages a Transformer to model spatial interactions among pedestrians. Spatial attention is employed to refine point cloud features by emphasizing pedestrian point cloud correlations, while modal attention fuses pedestrian position and point cloud representations to enhance predictive accuracy.

A multi-feature extraction-based compression technique is proposed to achieve efficient lossy point cloud compression. This method mitigates the spatial complexity of FlatFormer [10] positional encoding by applying coordinate decomposition principles. Three categories of point cloud features are extracted and fused to yield richer feature representations, thereby improving compression fidelity.

Extensive experiments are conducted from the perspective of point cloud compression and downstream trajectory prediction. The results demonstrate that the proposed framework achieves effective point cloud compression and that the compressed point cloud representations can support competitive performance in multimodal trajectory prediction.

The rest of the paper is organized as follows: Section 2 reviews related work on pedestrian trajectory prediction and point cloud compression. Section 3 introduces the preliminaries. Section 4 describes the proposed CPCAE framework in detail, covering the MF-Depoco module, attention mechanisms, and the encoder–decoder architecture. Section 5 presents experimental settings, performance analysis, and ablation studies. Finally, Section 6 concludes the paper and outlines future research directions.

## 2. Related Work

Early studies on pedestrian trajectory prediction primarily relied on historical trajectory data, employing recurrent neural networks (RNNs), long short-term memory networks (LSTMs), and their variants to model temporal dependencies within motion sequences [11]. However, these unimodal approaches often fail to capture complex scene constraints and highly dynamic pedestrian interactions [12], leading to limited prediction accuracy in real-world environments. To address these challenges, recent research has increasingly incorporated multimodal information such as video imagery, high-definition maps, and LiDAR point clouds. Among these, point cloud data precisely characterize pedestrians’ three-dimensional spatial positions and geometric structures while preserving privacy by avoiding exposure of sensitive biometric attributes such as individual identities.

Building on the remarkable success of the Transformer architecture in temporal and visual modeling, researchers have recently adapted it to sparse three-dimensional point cloud data to capture long-range dependencies and complex geometric relationships [13]. For example, ScatterFormer [14] pioneers the direct application of attention to voxels across different windows, treating them as a single sequence. Its cross-window interaction module enhances both locality and connectivity among voxel features, thereby eliminating the need for extensive window shifting. Similarly, Point Transformer V3 [15] adopts a serialization-based mechanism to map 3D points into 1D sequences. By utilizing serialized neighbor search and grouped vector self-attention, it achieves high scalability and efficiency on large-scale point clouds while preserving effective spatial interactions. Despite these advances, the high-dimensional sparsity of point cloud data remains a major obstacle to deploying Transformer-based models on resource-constrained in-vehicle platforms.

Addressing data redundancy in point clouds requires the development of highly efficient compression techniques. Traditional approaches, such as octree-based coding [16], often exhibit substantial geometric distortion at low bit rates. In contrast, deep learning-driven point cloud compression methods employ end-to-end learning frameworks and have demonstrated superior rate–distortion performance over conventional codecs, thereby emerging as the predominant research paradigm [17]. Existing studies primarily concentrate on two directions: enhancing rate–distortion efficiency and improving the compression of point cloud sequences. For example, Liu et al. [18] proposed a multi-stage space-to-channel context model capable of processing both dense and sparsely sampled point clouds. By incorporating geometric residual coding, this method enables cross-hierarchical prediction at consistent resolutions, effectively mitigating resolution constraints. To further improve coding efficiency, RENO [19] proposed a real-time neural compression framework for 3D LiDAR point clouds, which reduces the complexity of hierarchical geometry coding and enables low-latency compression through sparse-convolution-based occupancy and coordinate generation. For dynamic point clouds, Shao et al. [20] proposed an advanced patch-based affine motion estimation scheme that explicitly modeled inter-frame motion information, thereby improving temporal compression performance; however, its motion estimation module degrades in scenarios involving dense pedestrian interactions and complex motion patterns.

However, existing methods mainly emphasize geometric reconstruction while overlooking downstream task performance, making it difficult to preserve the semantic and interaction information required for trajectory prediction. To address this issue, CPCAE extracts and fuses semantic and spatial features before encoding, enabling compressed representations to remain task-relevant even at high compression ratios. Meanwhile, an attention-enhanced mechanism further improves feature representation by dynamically modeling pedestrian–scene interactions in compressed point cloud scenes. As a result, CPCAE maintains high trajectory prediction accuracy while balancing compression efficiency and reconstruction quality, providing strong support for task-oriented point cloud compression research.

## 3. Preliminaries

The Symmetric Point-to-Point Chamfer Distance (SCD) alleviates the bias induced by differences in point cardinality and emphasizes point-wise correspondence, thus providing a more reliable measure of the structural similarity between two point clouds. It is adopted to assess the reconstruction quality of compressed point clouds and is formulated:

(1) $$SCD_{sym}(PC,PC^{\prime })=\frac{\sum_{pc\in PC}\underset{pc^{\prime }\in PC^{\prime }}{\min}{\left\|pc-pc^{\prime }\right\|}_{2}^{2}}{\left|PC\right|}+\frac{\sum_{pc^{\prime }\in PC^{\prime }}\underset{pc\in PC}{\min}{\left\|pc-pc^{\prime }\right\|}_{2}^{2}}{\left|PC^{\prime }\right|}$$

where PC and $PC^{\prime }$ denote the original and reconstructed point cloud sets, respectively, pc and $pc^{\prime }$ represent points in PC and $PC^{\prime }$.

## 4. CPCAE Method

The point cloud is a geometric representation of a three-dimensional object or scene composed of discrete sampled points. The 3D LiDAR pedestrian point cloud used in this paper is defined as a finite set of points $PC=\{pc_{1},pc_{2},\ldots ,pc_{\left|PC\right|}\}$, where $pc_{i}$ denotes an individual point with its three-dimensional spatial coordinates $pc_{i}(x_{i},y_{i},z_{i})$.

Pedestrian bounding boxes from the forward camera perspective are annotated using images captured by the same camera to indicate pedestrian positional information. Each bounding box can be defined by its top-left and bottom-right coordinates, expressed as $bbox(x_{\min},y_{\min},x_{\max},y_{\max})$, specifically, $x_{\min}$ and $y_{\min}$ denote the coordinates of the top-left corner, $x_{\max}$ and $y_{\max}$ denote the coordinates of the bottom-right corner.

Based on these definitions, this paper employs a pedestrian trajectory dataset $D_{ped}$ as input, which contains a 3D LiDAR pedestrian point cloud and forward camera pedestrian bounding boxes. A fixed number of time steps from this dataset is used as input to the proposed method to predict pedestrian trajectories of a specified length.

This section presents the compressed point cloud and attention-enhanced trajectory prediction method. The proposed method compresses the point cloud and employs attention mechanisms to enhance and fuse pedestrian position features with point cloud features, providing a new solution to the challenges of accuracy and efficiency in pedestrian trajectory prediction. Section 4.1 introduces the overall framework of CPCAE. Section 4.2.1 and Section 4.2.2 describe the MF-Depoco module. Section 4.2.3 presents the implementation of two modality processing. Section 4.3 describes the related attention mechanisms, and Section 4.4 details the decoder design and formulates the objective function.

### 4.1. Overall Architecture

**Figure 1:**
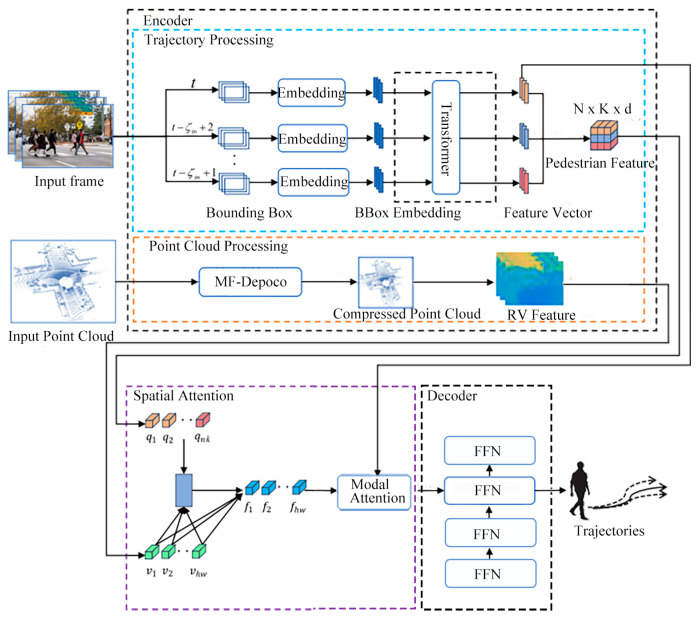
The CPCAE method framework. Pedestrian bounding boxes and point cloud data are encoded into motion and range-view features, respectively. These features are fused by spatial–modal attention and fed into fully connected layers to predict future pedestrian trajectories.

Figure 1 presents the framework of the proposed method. The CPCAE method adopts an encoder–decoder architecture consisting of three primary parts: the encoder, the attention mechanism, and the decoder.

The trajectory encoder embeds historical pedestrian bounding boxes and feeds them into a Transformer to encode pedestrian features. In parallel, the point cloud encoder first extracts diverse point cloud features through a multi-feature extraction module and then performs point cloud compression. This overall process constitutes the MF-Depoco module. Subsequently, the compressed point cloud is transformed into a range-view representation and processed by 2D convolutions to obtain RV features.

The attention mechanism comprises spatial and modal attention submodules. The former captures spatial correlations between pedestrian position features and RV image features, emphasizing the regions of the RV image most relevant to each pedestrian’s position. The latter identifies and strengthens the inter-modal correlations between the RV image and pedestrian position features, integrating the weighted aggregation as a pedestrian-specific context feature with the pedestrian position features. Finally, the fused features are fed into fully connected layers to predict pedestrian trajectories.

### 4.2. Encoder

#### 4.2.1. Lossy Point Cloud Compression

Lossy point cloud compression reduces data volume by removing redundant information while preserving the global geometry and structural integrity of the point cloud, thereby producing a compact representation for storage and transmission. The point cloud compression module is an improvement of the lossy compression framework Depoco [21].

This module integrates a multi-feature extraction component, which enhances the FlatFormer and collaborates with both SPoTransformer (Self-Positioning point-based Transformer) [22] and density embedding mechanism to extract diverse point cloud features, which is described in detail in the following sub-Section 4.2.2. These extracted features are then fed into the Depoco encoder to improve its representational capacity for better compression. The Depoco encoder employs down-sampling to reduce point cardinality while preserving essential information. The Depoco decoder progressively reconstructs the point cloud through up-sampling, ultimately producing the compressed point cloud.

Depoco Encoder. For the input dataset of raw point cloud $PC_{raw}=\{pc_{i})|i=1,2,\ldots ,\left|PC_{raw}\right|\}$, it is processed by the multi-feature extraction component to generate voxel-level feature $f_{i}$ for each point $pc_{i}$, and the dataset $PC_{in}=\{(pc_{i},f_{i})|i=1,2,\ldots ,\left|PC_{in}\right|\}$ is produced. To reduce the cardinality of the point cloud while preserving its essential characteristics, a subset is generated via grid-based down-sampling. Kernel Point Convolution (KPConv) [23] is then applied to directly aggregate point-level features, thereby mitigating the drawbacks of voxel-level feature aggregation. For the dataset $PC_{in}$, three successive iterations of grid-based down-sampling and KPConv is executed, ultimately producing a compressed point cloud embedding representation through MLP.

Depoco Decoder. The Depoco decoder reconstructs the raw point cloud from its embedding representation through a four-layer deconvolutional architecture, which progressively upsamples the compact representations to restore the original point cloud density. By employing a multi-stage feature propagation strategy, the Depoco decoder mitigates the discretization error commonly associated with the voxelization approach. Moreover, it achieves high-fidelity reconstruction at a high compression rate without the need for a skip connection.

#### 4.2.2. Multi-Feature Extraction for Point Cloud

**Figure 2:**
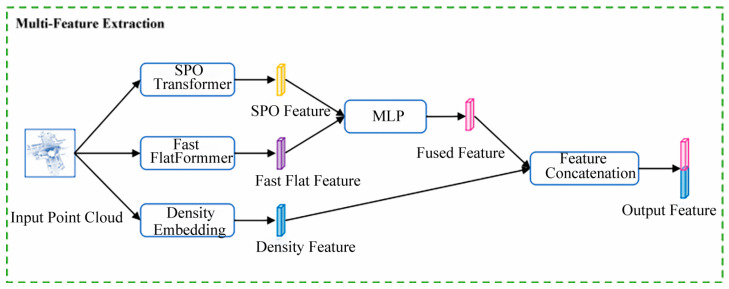
The multi-feature exaction component. The input point cloud is processed by the SPOTransformer, Fast FlatFormer, and density embedding module to extract complementary features. These features are fused and concatenated to generate the final point cloud representation.

The multi-feature extraction component incorporates three distinct categories of point cloud features, and the design of this component is illustrated in Figure 2.

The SPOTranformer extracts spatial and semantic features from the point cloud. The Fast FlatFormer enhances FlatFormer by primarily leveraging coordinate decomposition and refines both the positional encoding and attention mechanisms of FlatFormer to extract detailed point cloud features. The density embedding part captures the spatial distribution and density characteristics of the point cloud through density embedding techniques. Finally, the three types of features are fused to generate the final output representation.

**Figure 3:**
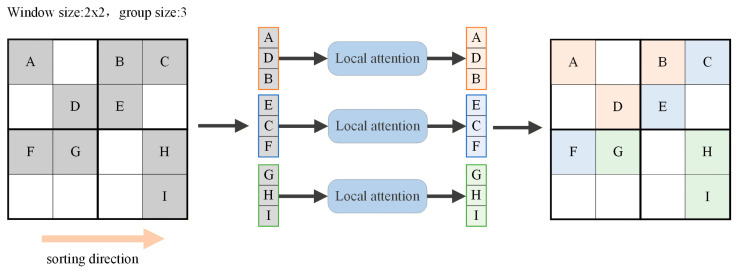
The partition strategy and attention of Fast Flat Former. Input features are first partitioned into spatial windows, sorted by position, and organized into fixed-size groups. Local attention is then applied within each group, and the updated features are restored to their original spatial layout to obtain enhanced point cloud representations.

Fast Flat Former. Based on FlatFormer architecture, Fast FlatFormer adopts the coordinate decomposition strategy [24] to reduce the spatial complexity of positional encoding. The core principle of coordinate decomposition involves decomposing the relative positions of voxel center points:

(2) $$cen_{i}-cen_{j}=\left(cen_{i}-vox_{i}\right)-\left(cen_{j}-vox_{j}\right)+\left(vox_{i}-vox_{j}\right)$$

where $cen_{i}$ and $cen_{j}$ denote the center coordinates of the voxel, $vox_{i}$ and $vox_{j}$ represent the coordinates of the corresponding voxel. The spatial complexity of the position encoding $\phi_{\mathrm{abs}}\left(cen_{i}-cen_{j}\right)$ is $O(I\cdot num_{K}\cdot num_{dim})$, where I denotes the total number of voxels, $num_{K}$ represents the maximum number of neighbors, and $num_{dim}$ indicates the encoding dimension. The position encoding $\phi_{\mathrm{abs}}\left(cen_{i}-cen_{j}\right)$ can be decomposed into two types: continuous position encoding $\phi_{\mathrm{abs}}\left(cen_{i}-vox_{i}\right)$ and discrete position encoding $\phi_{\mathrm{rel}}\left(vox_{i}-vox_{j}\right)$. The space complexity of the continuous position encoding $\phi_{\mathrm{abs}}\left(cen_{i}-vox_{i}\right)$ is $O(I\cdot num_{dim})$. Notably, the discrete position encoding $\phi_{\mathrm{rel}}\left(vox_{i}-vox_{j}\right)$ is memory-efficient, since for all possible $\left(vox_{i}-vox_{j}\right)$, there exist only K distinct discrete relative positions. Consequently, the space complexity of $\phi_{\mathrm{abs}}\left(cen_{i}-cen_{j}\right)$ is reduced from $O(I\cdot num_{K}\cdot num_{dim})$ to $O(I\cdot num_{dim}+num_{K}\cdot num_{dim})$. The grouping strategy and local self-attention mechanism of Fast FlatFormer are illustrated in Figure 3.

Fast FlatFormer implements local self-attention through the following procedure, by leveraging the coordinate decomposition strategy. Given a voxel set $S_{Vox}$ derived from point cloud data, the initial window-based sorting process transforms the unordered point cloud into an ordered representation. Specifically, all voxels within each window are partitioned into equal-sized groups based on sorting along either the *x*-axis or the *y*-axis. For each voxel within a group, both a continuous position encoding $\phi_{\mathrm{abs}}$ and a discretized position encoding $\phi_{\mathrm{rel}}$ are generated. The local self-attention for each voxel is then computed as follows:

(3) $$f_{vox,i}^{\prime }=\underset{j\in Nei(i)}{\sum }\frac{\psi_{trans1}\left(g_{vox,i}\right)\cdot \phi_{\mathrm{rel}}\left(vox_{i}-vox_{j}\right)}{∥\psi_{trans1}\left(g_{vox,i}\right)∥\times ∥\phi_{\mathrm{rel}}\left(vox_{i}-vox_{j}\right)∥}\psi_{trans2}\left(ge_{j}\right)$$

where $f_{vox,i}^{\prime }$ denotes the output feature of the i-th voxel, Nei(i) represents the set of neighboring voxels for the i-th voxel, and $g_{vox,i}=f_{vox,i}+\delta_{\mathrm{abs}}\left(c_{i}-v_{i}\right)$ is the aggregated feature obtained by the consecutive position encoding of the i-th voxel. $f_{vox,i}$ denotes the input feature of the i-th voxel. $\psi_{trans1}$ and $\psi_{trans2}$ are learnable transformation functions. Point cloud data are fed into the Fast FlatFormer to extract features $f_{Fast}$ for each point in a voxel.

SPOTranformer. SPOTransformer [22] utilizes a local self-attention mechanism and a global cross-attention mechanism based on self-positioning points, effectively capturing both spatial and semantic information. To enrich the feature representation and achieve a more comprehensive understanding of the point cloud, the feature $f_{SPo}$ produced by SPOTransformer is subsequently fused with the feature $f_{Fast}$ from the Fast FlatFormer via MLP to generate the combined feature $f_{SF}\in R^{num_{vox}\times C_{SF}}$:

(4) $$f_{SF}=\mathrm{MLP}([f_{SPo};f_{fast}])$$

In the above formula, $\left[f_{SPo};f_{Flat}\right]$ denotes the connection of $f_{SPo}\in R^{num_{vox}\times C_{SPO}}$ and $f_{Fast}\in R^{num_{vox}\times C_{fast}}$ along the feature dimension. Here, $num_{vox}$ denotes the number of points contained in the voxel, and $C_{SPO}$, $C_{Fast}$, and $C_{SF}$ denote the corresponding feature channel dimensions.

Density Embedding. Furthermore, to effectively capture the spatial distribution and density characteristics of points within a voxel, density embedding [25] is utilized to extract representative features. For each point $pc_{i}$ within the voxel centered at cen, the angular and distance-based offsets from the voxel center are computed:

(5) $$\left(\frac{pc_{i}-cen}{{\left\|pc_{i}-cen\right\|}_{2}},{\left\|pc_{i}-cen\right\|}_{2}\right)$$

The direction (three-dimensional) and distance (scalar) between each point and its corresponding voxel center are jointly represented as a four-dimensional vector. Accordingly, the spatial distribution of points within the voxel can be characterized by a feature $num_{vox}\times 4$, where $num_{vox}$ denotes the total number of points contained in the voxel. This feature is then projected into a higher-dimensional latent space through MLP, producing the density embedding feature $f_{Dens}\in R^{1\times C_{Dens}}$. Specifically, the voxel-level density feature $f_{Dens}$ is repeated $num_{vox}$ times along the point dimension, such that each point in the voxel receives the same density embedding. The broadcasting operation is formulated as ${f_{Dens}}^{\prime }=\mathrm{Broadcast}(f_{Dens})\in R^{num_{vox}\times C_{Dens}}$, where $C_{Dens}$ denotes the channel dimension of the density embedding feature, and ${f_{Dens}}^{\prime }$ denotes the broadcast point-level density feature.

Finally, the above fused features $f_{SF}$ and ${f_{Dens}}^{\prime }$ are concatenated to obtain the final output feature $f\in R^{num_{vox}\times (C_{SF}+C_{Dens})}$:

(6) $$f=([f_{SF};{f_{Dens}}^{\prime }])$$

#### 4.2.3. Two Modality Processing

The encoder consists of two primary components: point cloud processing and trajectory processing.

Point cloud processing. A common approach for representing 3D LiDAR point clouds is to project them onto a 2D Range View image. The RV image provides a compact yet informative representation that preserves the local geometric structure of the LiDAR data. In the point cloud processing component, the raw point cloud data is compressed by the lossy compression module, and then subsequently projected into a 2D RV image. A 2D convolutional network is applied to derive the feature matrix from the generated RV image. Before convolution, the input RV image is padded to maintain spatial consistency, after which it is passed through the convolutional layer to produce the final RV feature map $F_{RC}$.

Trajectory processing. The trajectory processing component employs a learnable embedding to map pedestrian bounding boxes across multiple time steps into a latent representation, which is subsequently processed by a Transformer to capture spatiotemporal position features.

To effectively capture temporal dependencies in pedestrian motion, the bounding boxes within $\zeta_{in}$ consecutive time steps are input to the Transformer. Given the current time step t, the model incorporates both the current pedestrian bounding box input $B_{t}$ and the historical bounding box input $B_{t-\zeta_{in}+1},B_{t-\zeta_{in}+2},\ldots ,B_{t-1}$ from the $t-\zeta_{in}+1,t-\zeta_{in}+2,\ldots ,t-1$ time step. By integrating historical bounding box sequences, the model can accurately represent pedestrian motion patterns.

Specifically, each pedestrian bounding box $B_{i}$ is projected into a high-dimensional space via a learnable embedding, yielding the overall pedestrian positional embedding $e_{B}$. For notational simplicity, the temporal index is omitted in Equations (7)–(10), where i denotes the pedestrian index, equivalent to n used in the following attention mechanism; nevertheless, the resulting feature $F_{bbox}$ still preserves the observed temporal dimension:

(7) $$e_{bbox}^{i}=\varpi \left(W_{bbox}\cdot B_{i}\;+\;bias_{bbox}\;\right)$$

(8) $$e_{B}=\left(e_{bbox}^{1},e_{bbox}^{2},\ldots ,e_{bbox}^{N_{ped}}\right)$$

where ϖ denotes the ReLU nonlinear activation function, $W_{bbox}$ is the learnable parameter matrix for embedding, $bias_{bbox}$ represents the bias term, and $e_{bbox}^{i}$ denotes the embedding of the i-th pedestrian bounding box. $e_{B}$ represents the set of pedestrian bounding box embeddings.

The Transformer is capable of effectively processing multiple pedestrian bounding boxes across consecutive time steps and modeling the spatial interactions among pedestrians. Specifically, the pedestrian bounding box embedding set $e_{B}$ at each time step is combined with the positional encoding and then fed into the Transformer to obtain pedestrian positional features:

(9) $$F_{bbox}=\mathrm{Transformer}(e_{B}+\phi_{bbox})$$

(10) $$F_{bbox}=\left(f_{bbox}^{1},f_{bbox}^{2},\ldots ,f_{bbox}^{N_{ped}}\right)$$

where $F_{bbox}$ denotes the overall positional features of all pedestrians, $f_{bbox}^{i}$ denotes the positional feature of the i-th pedestrian, and $\phi_{bbox}$ denotes the corresponding positional encoding.

### 4.3. Attention Mechanism

The attention mechanism in the proposed method consists of two parts: spatial attention and modal attention. The spatial attention module captures the spatial relationship between pedestrian position features and RV image features, emphasizing the regions of the RV image that are most relevant to pedestrian positions. The modal attention module focuses on salient regions of the RV image and pedestrian position features over the observed input sequence, integrating their weighted representations into pedestrian-specific context features for subsequent fusion.

Spatial Attention. The spatial attention module adopts a cross-attention formulation, using pedestrian position features as queries and RV image features as keys and values.

Specifically, matrix $Q(Q\in R^{N_{ped}\times \zeta_{in}\times d_{fbbox}})$ is constructed by stacking bounding box vectors refined by the Transformer over $\zeta_{in}$ time steps, where $d_{fbbox}$ denotes the dimensionality of each vector and $N_{ped}$ is the number of pedestrians. matrix Q encodes the spatial information of pedestrian targets, corresponding to the positions within the pedestrian bounding boxes. Matrix K represents the RV image feature $F_{RC}$ after convolutional processing, with feature dimensions $Hig\times Wid\times d_{RC}$, where Hig, Wid and $d_{RC}$ denote the height, width, and channel dimension, respectively. The RV image feature, denoted as matrix K, captures the spatial contextual cues that facilitate understanding of the pedestrian’s position within the scene. Finally, matrix V, derived from the same RV feature map, provides the scene features to be aggregated according to the attention weights computed from Q and V, producing the spatial attention output $F_{\mathrm{RV}\text{-}\mathrm{att}}\in R^{N_{\mathrm{ped}}\times \zeta_{\mathrm{in}}\times d_{\mathrm{RC}}}$. The feature set $F_{\mathrm{RV}\text{-}\mathrm{att}}$ contains pedestrian-specific RV features, where each vector is enhanced according to its corresponding pedestrian bounding box query.

The spatial attention is computed as follows. We first traverse the query matrix Q along its first and second dimensions, yielding $N_{ped}\times \zeta_{in}$ query vectors $q_{nk}\in R^{d_{fbbox}}$, where nk=(n,k) denotes the pedestrian-time index pair, where $n=1,\ldots ,N_{ped}$ and $k=1,\ldots ,\xi_{in}$. For each query vector $q_{nk}$, its similarity to the key vectors in K is computed. By traversing K row-wise, the RV feature map is flattened into Hig × Wid RV key-feature vectors $k_{hw}$, where hw=(h,w) denotes the RV spatial index pair, where h=1,…,Hig and w=1,…,Wid. Meanwhile, by traversing V row-wise, the same RV feature map is flattened into Hig × Wid value vectors $v_{hw}$. The attention score ${\mathrm{score}}_{nk}^{hw}$ is then computed by the dot product between $q_{nk}$ and $k_{hw}$:

(11) $${\mathrm{score}}_{nk}^{hw}={\left(W_{\mathrm{spa}}k_{hw}\right)}^{T}q_{nk}$$

where $W_{spa}$ denotes the weight matrix of the linear layer, which aligns the dimensionality of $k_{hw}$ with that of $q_{nk}$.

The attention scores ${\mathrm{score}}_{nk}^{hw}$ between $q_{nk}$ and the key-feature vectors $k_{hw}$ at all Hig×Wid locations are computed and normalized by the Softmax function to obtain the attention weights $\alpha_{nk}^{hw}$. Subsequently, the attention weights $\alpha_{nk}^{hw}$ are used to aggregate the feature vectors $v_{hw}$ through weighted summation:

(12) $$\alpha_{nk}^{hw}={\mathrm{Softmax}}_{h,w}\left({\mathrm{score}}_{nk}^{hw}\right)$$

(13) $${\hat{v}}_{nk}=\sum_{h=1}^{Hig}\sum_{w=1}^{Wid}\alpha_{nk}^{hw}v_{hw}$$

After all query vectors $q_{nk}$ have been processed, the obtained vectors ${\hat{v}}_{nk}$ are arranged according to the traversal order of Q to form the spatial attention output $F_{RV-att}$. Therefore, $F_{RV-att}\in R^{N_{ped}\times \zeta_{in}\times d_{RC}}$. The output $F_{\mathrm{RV}\text{-}\mathrm{att}}$ contains enhanced RV features, where each vector ${\hat{v}}_{nk}$ is aggregated from RV regions most relevant to the corresponding pedestrian bounding box query.

Modal Attention. To integrate the spatial attention output $F_{\mathrm{RV}\text{-}\mathrm{att}}$ with the enhanced pedestrian bounding box features $F_{\mathrm{bbox}}$ over the observed input sequence, the proposed method adopts a modal attention mechanism. This mechanism measures the correlations between each pedestrian feature $f_{\mathrm{bbox}}^{i}$ and the spatially enhanced RV features ${\hat{v}}_{nk}$. For the i-th pedestrian, the modal attention weights are used to aggregate the spatially enhanced RV features into a pedestrian-specific weighted feature $A_{i}$. Then, $A_{i}$ is concatenated with $f_{\mathrm{bbox}}^{i}$ and passed through a feed-forward neural network (FFN) to obtain the modal-attended feature $f_{\mathrm{modal}\text{-}\mathrm{att}}^{i}$. Finally, the modal-attention feature set $F_{\mathrm{modal}\text{-}\mathrm{att}}$ is obtained.

The modal attention is computed as follows. The spatial attention output $F_{\mathrm{RV}\text{-}\mathrm{att}}$ is first reorganized into a feature-vector set composed of ${\hat{v}}_{nk}$. For each pedestrian feature $f_{\mathrm{bbox}}^{i}$, a similarity function sim is used to compute the modal-attention weights between $f_{\mathrm{bbox}}^{i}$ and each spatially enhanced RV feature ${\hat{v}}_{nk}$:

(14) $$\mathrm{sim}\left(f_{\mathrm{bbox}}^{i},{\hat{v}}_{nk}\right)={\left(f_{\mathrm{bbox}}^{i}\right)}^{T}W_{\mathrm{sim}}{\hat{v}}_{nk}$$

(15) $$\alpha_{i,nk}^{\mathrm{modal}}={\mathrm{Softmax}}_{n,k}\left(\mathrm{sim}\left(f_{\mathrm{bbox}}^{i},{\hat{v}}_{nk}\right)\right)$$

where $W_{\mathrm{sim}}$ is a learnable parameter matrix. For the i-th pedestrian, the modal-attention weights $\alpha_{i,nk}^{\mathrm{modal}}$ are applied to the spatially enhanced RV feature set composed of ${\hat{v}}_{nk}$ to obtain the pedestrian-specific weighted feature $A_{i}$:

(16) $$A_{i}=\sum_{n=1}^{N_{\mathrm{ped}}}\sum_{k=1}^{\zeta_{\mathrm{in}}}\alpha_{i,nk}^{\mathrm{modal}}{\hat{v}}_{nk}$$

**Figure 4:**
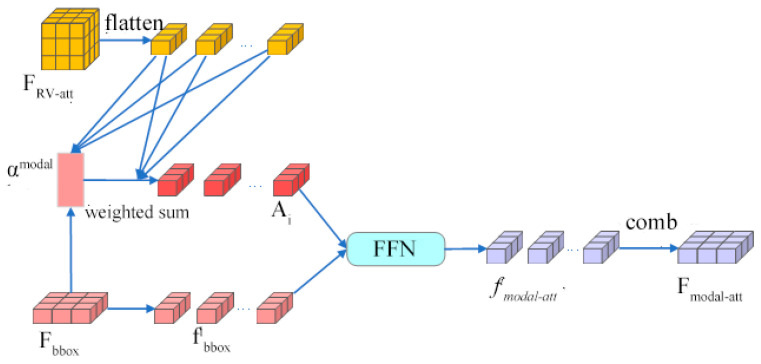
Modal attention. This module models the correlations between refined RV image features and pedestrian bounding box features over the observed input sequence. The features are fused through modal-attention weighting and feed-forward processing to generate the final modal-attended representation.

The weighted feature $A_{i}$ is concatenated with the bounding box feature $f_{\mathrm{bbox}}^{i}$ of the same pedestrian and then fed into a feed-forward network to generate the modal-attended feature $f_{\mathrm{modal}\text{-}\mathrm{att}}^{i}$ for the i-th pedestrian. The resulting features are further organized into the modal-attention feature set $F_{\mathrm{modal}\text{-}\mathrm{att}}$:

(17) $$f_{\mathrm{modal}\text{-}\mathrm{att}}^{i}=\tanh\left(W_{\mathrm{modal}}\left[A_{i};f_{\mathrm{bbox}}^{i}\right]\right)$$

(18) $$F_{\mathrm{modal}\text{-}\mathrm{att}}=Stack\left(f_{\mathrm{modal}\text{-}\mathrm{att}}^{1},f_{\mathrm{modal}\text{-}\mathrm{att}}^{2},\ldots ,f_{\mathrm{modal}\text{-}\mathrm{att}}^{N_{\mathrm{ped}}}\right)$$

where tanh denotes the hyperbolic tangent activation function, and $W_{\mathrm{modal}}$ represents the parameters of the FFN. Modal attention is illustrated in Figure 4. After attention processing, a set of modal attention feature vectors is generated and subsequently fed into the decoder to predict the pedestrian bounding box at the next time step.

### 4.4. Decoder

The decoder utilizes four different fully connected layers to process the feature vector set $F_{model\text{-}att}$ derived from the modal attention and predicts the pedestrian bounding box coordinates for the subsequent time step. The bounding boxes are reformulated into a center-based representation. Specifically, each predicted bounding box is expressed by its center point coordinates and corresponding width and height.

The proposed method uses a fixed-length input sequence to predict the bounding box for the subsequent time step. The output bounding box is then recursively fed back as input to generate predictions for the following time steps. This iterative procedure allows to forecast pedestrian trajectories over $\zeta_{out}$ time steps.

To evaluate and optimize the performance of the CPCAE method, its loss function $L_{pred}=L_{pc}+\lambda L_{bbox}$ is decomposed into two parts: the point cloud compression loss $L_{pc}$ and the pedestrian bounding box prediction loss $L_{bbox}$. λ is a balancing coefficient that controls the relative contribution of the two loss terms. For the first part, according to the Symmetric Point-to-Point Chamfer Distance (SCD), the loss function associated with the Depoco model is adopted:

(19) $$L_{pc}=\frac{1}{2}(SCD_{sym}(PC,PC^{\prime })+\beta \sum_{j}SCD_{sym}(PC,\hat{P}C_{j}))$$

where PC and $PC^{\prime }$ denote the original and reconstructed point clouds, respectively, $\hat{P}C_{j}$ denotes the output point set of the j-th deconvolution module in Depoco, and β signifies the weight control factor of the regularization term.

For the second part, the IoU loss $L_{\mathrm{IoU}}$ is adopted as the loss function $L_{bbox}$ for the predicted pedestrian trajectory:

(20) $$L_{bbox}=\frac{1}{N_{ped}\zeta_{out}}\sum_{i=1}^{N_{ped}}\sum_{t=1}^{\zeta_{out}}\left(1-\frac{\left|{\hat{bbox}}_{i}^{t}\cap bbox_{i}^{t}\right|}{\left|{\hat{bbox}}_{i}^{t}\cup bbox_{i}^{t}\right|}\right)$$

where $N_{ped}$ denotes the number of pedestrians in the scene, $\zeta_{out}$ is the number of future prediction steps, ${\hat{bbox}}_{i}^{t}$ and $bbox_{i}^{t}$ denote the predicted and ground-truth bounding boxes of the i-th pedestrian at the t-th prediction step, respectively. The IoU loss is employed to minimize the discrepancy between the predicted and ground-truth bounding boxes, thereby improving the accuracy of trajectory prediction.

## 5. Experiments

This section first outlines the experimental environment, evaluation metrics, and parameter configurations used to implement the CPCAE method. Subsequently, comparative experiments are conducted, and their results are thoroughly analyzed.

### 5.1. Experimental Settings

Experimental Environment. The experimental setup consists of an Intel i7-10700KF @3.80 GHz CPU, NVIDIA GeForce 4090 Ti, and a 64-bit Windows 10 operating system. The implementation is developed in Python 3.10 using PyTorch 2.6.0.

Implementation Details. For trajectory prediction, the encoder takes historical pedestrian bounding box sequences as input, which are normalized according to the image resolution before being fed into the network. Each bounding box at each time step is first embedded into a 256-dimensional feature space through a linear projection and then combined with temporal positional encoding. A two-layer Transformer encoder is subsequently used to model temporal dependencies in historical bounding box sequences. Each Transformer layer contains four attention heads, with the hidden dimension of the feed-forward network set to 512, dropout set to 0.1, and ReLU used as the activation function. In the MF-Depoco compression module, the input point cloud is first sparsified with a voxel resolution of 0.1 m and then fed into the multi-feature extraction branches. In the SPOTransformer branch, the number of sampled local neighbors is set to 32, and the number of self-positioning points is set to 16. The Fast FlatFormer branch extracts features using grouped attention within local windows, with the input voxel resolution kept consistent with the point cloud preprocessing. The density branch uses local point-count statistics as density descriptors and maps them to density features through an MLP. The compression backbone adopts a three-layer GridSampleConv encoder and a four-layer AdaptiveDeconv decoder. In the encoder, the number of KPConv kernel points is set to 27, and the maximum numbers of neighboring points are set to 70, 50, and 25 for the three encoding layers, respectively. The encoder outputs a compact point cloud embedding. In the decoder, the intermediate feature dimension is set to 128, and the upsampling kernel radius is set to 0.05. During inference, K candidate trajectories are sampled for each input, and a best-of-K strategy is adopted to compute the Average Displacement Error (ADE), Center Average Displacement Error (CADE), and Center Final Displacement Error (CFDE). All reported results are averaged over five random seeds.

Training Settings. A staged training strategy is adopted in this study. In the first stage, the MF-Depoco point cloud compression module is trained independently to learn stable geometric compression and reconstruction representations. This stage follows the Depoco backbone training configuration, using the Adam optimizer and a OneCycle learning-rate schedule, with the maximum learning rate set to $1\times 10^{-4}$. Each mini batch contains three point cloud submaps, and the number of training epochs is set to 200. The loss function consists of a bidirectional Chamfer reconstruction loss and an upsampling regularization term, which jointly constrain the geometric consistency between the reconstructed and original point clouds and the stability of the upsampling process.

In the second stage, given that SemanticKITTI is distributionally closer to KITTI and SHIFT, the second stage initializes MF-Depoco with weights pretrained on SemanticKITTI. MF-Depoco is then integrated into the CPCAE framework, and the entire model is jointly optimized on the corresponding dataset. During training, MF-Depoco, the trajectory encoder, the attention modules, and the decoder are updated simultaneously, enabling the compression module to adapt to the target domain within the downstream task training process. For trajectory prediction, the Adam optimizer is used, with the initial learning rate set to $1\times 10^{-3}$, the batch size set to 128, and the number of training epochs set to 300. The model operates in the normalized coordinate space during both training and inference. During evaluation, the predicted results are denormalized back to pixel coordinates, and ADE, CADE, and CFDE are computed.

Two groups of comparative experiments are conducted. The first group evaluates the reconstruction quality of different point cloud compression methods under the same bits-per-point (BPP) setting using Symmetric Point-to-Point Chamfer Distance and Point-to-Plane PSNR. The second group evaluates the trajectory prediction performance of CPCAE against existing methods on the KITTI and SHIFT datasets. In addition, ablation studies are conducted to validate the effectiveness of MF-Depoco, spatial attention, and modal attention in the overall framework.

### 5.2. Analysis of Point Cloud Compression Experiments

The designed lossy point cloud compression module is evaluated on the SemanticKITTI [26] and ShapeNet [27] datasets. The SemanticKITTI dataset includes 22 sequences and is divided according to the official training and test set division, where sequences between 00 and 10 are used for training and sequences between 11 and 21 for testing. The training set and the test set contain 23,201 and 20,351 LiDAR point cloud samples, respectively. The ShapeNet dataset consists of LiDAR point clouds from 16 object categories. Following the training/test set division of Huang et al. [28], the training set and the test set contain 12,288 samples and 2874 samples, respectively. And we adopt the point sampling strategy for the voxel grid given by Hermosilla et al. [29].

Our lossy compression module is evaluated against the following representative point cloud compression approaches. The reconstruction quality is comprehensively analyzed under the condition of bits per point (BPP).

Google Draco [30] is an open-source 3D point cloud compression library, which employs geometric compression and topological encoding to reduce storage and transmission cost while maintaining high-fidelity geometric structures. MPEG Anchor [31] serves as a general-purpose, real-time point cloud codec that improves compression efficiency and perceptual quality by integrating octree encoding, inter-frame prediction, and JPEG-based color compression techniques. Depoco [21] is a deep convolutional autoencoder that utilizes KPConv layers to reconstruct point clouds efficiently without voxelization.

This paper adopts bits per point (BPP) as a unified measure of point cloud coding rate. Given an input point cloud PC with $N_{pts}$ points, BPP is defined as the ratio between the total number of bits in the actual compressed bitstream and the number of input points:

(21) $$\mathrm{BPP}=\frac{B_{\mathrm{comp}}}{N_{\mathrm{pts}}}$$

Here, $B_{\mathrm{comp}}$ denotes the actual number of bits used to represent the point cloud in the compressed file. For Depoco and the proposed MF-Depoco, the encoder output consists of latent point coordinates and latent features, denoted by $Z_{p}$ and $Z_{f}$, respectively. To generate the compressed file, $Z_{p}$ and $Z_{f}$ are first converted into fixed-precision FP16 latent variables, denoted by ${\tilde{Z}}_{p}$ and ${\tilde{Z}}_{f}$. Standard lossless entropy coding is then applied to ${\tilde{Z}}_{p}$ and ${\tilde{Z}}_{f}$ to produce the corresponding compressed bitstream:

(22) $$B={\mathrm{Enc}}_{ent}({\tilde{Z}}_{p},{\tilde{Z}}_{f},H)$$

Here, ${\mathrm{Enc}}_{ent}(\cdot )$ denotes the lossless entropy encoding process, and H denotes the side information required for decoding, including tensor dimensions, the number of input points, data types, bitstream length, and file-header information. All these items are included in the compressed file size. Therefore, the actual BPP is computed as:

(23) $${\mathrm{BPP}}_{MF}=\frac{8S_{file}}{N_{pts}}$$

Here, $S_{file}$ denotes the compressed file size in bytes. This file size includes the entropy-coded latent point coordinates, latent features, and required side information. Since this work uses lossless entropy coding, the decoded latent variables remain identical to the FP16 latent variables before encoding. The entropy coding process only changes the storage format and bitstream size of the latent variables, without altering the latent contents fed into the decoder.

The reconstruction performance of the compressed point cloud is evaluated using the following two metrics: Symmetric Point-to-Point Chamfer Distance (SCD) and Point-to-Plane Peak Signal-to-Noise Ratio (Point-to-Plane PSNR). Point-to-Plane PSNR quantifies the error between the reconstructed point cloud and the original point cloud, which is calculated as follows:

(24) $$\mathrm{PSNR}(PC,PC^{\prime })=10\log_{10}\frac{\underset{i}{\max}{\left\|pc_{i}-pc_{i}^{\prime }\right\|}_{2}^{2}}{{\mathrm{MSE}}_{sym}(PC,PC^{\prime })}$$

where PC denotes the original point cloud, $PC^{\prime }$ denotes the reconstructed point cloud, and ${\mathrm{MSE}}_{sym}(PC,PC^{\prime })$ denotes the mean squared error for symmetric points relative to the plane:

(25) $$\mathrm{MSE}(PC,PC^{\prime })=\frac{1}{\left|PC\right|}{\sum_{i}\left((pc_{i}-pc_{i}^{\prime })\cdot n_{i}\right)}^{2}$$

(26) $${\mathrm{MSE}}_{sym}(PC,PC^{\prime })=\mathrm{MSE}(PC,PC^{\prime })+\mathrm{MSE}(PC^{\prime },PC)$$

where $n_{i}$ denotes the normal vector corresponding to the point $pc_{i}$.

**Figure 5:**
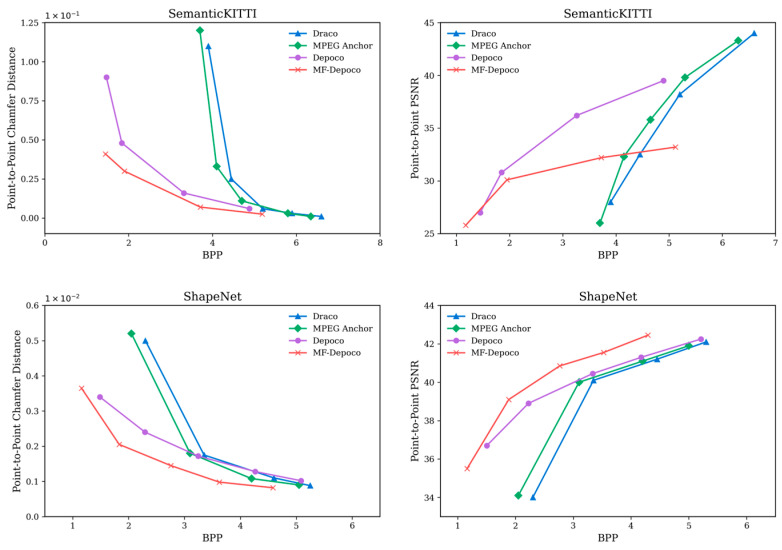
Comparison of point cloud reconstruction quality on two datasets. It presents a comparative analysis of SCD and Point-to-Plane PSNR across the above methods.

As illustrated in Figure 5, at an equivalent BPP, a lower SCD value and a higher Point-to-Plane PSNR value mean better point cloud reconstruction quality. Our lossy compression module achieves the lowest SCD value and the highest Point-to-Plane PSNR value on both SemanticKITTI and ShapeNet datasets, demonstrating its effectiveness in achieving more accurate reconstruction.

### 5.3. Analysis of Pedestrian Trajectory Prediction Experiments

Pedestrian trajectory prediction experiments were conducted on the KITTI [32] and SHIFT [33] datasets. Both datasets provide multimodal data for autonomous driving scenarios, including camera image frames, LiDAR point clouds, and corresponding object annotations. The KITTI dataset was collected from real-world road scenes and covers diverse traffic environments, including urban roads, vehicle interactions, and pedestrian motion. SHIFT is a synthetic autonomous driving dataset that provides vehicle and pedestrian data under varying weather, illumination, time-of-day, and scene-density conditions. To ensure consistency with existing camera-LiDAR-based multimodal trajectory prediction methods, this study adopts the same temporal settings as the reference method. For KITTI, consecutive frames sampled at 10 Hz are used: ten historical frames of bounding box observations within 1 s are taken as input, and pedestrian positions over the following 20 frames within 2 s are predicted. For SHIFT, consecutive frames sampled at 1 Hz are used: ten historical bounding box frames within 10 s are taken as input, and pedestrian positions over the following 20 frames within 20 s are predicted. Both datasets are split into training and test sets at the video-sequence level with an 80:20 ratio, and the same split is used for all comparative and ablation experiments.

The experiment adopted three widely used evaluation metrics for pedestrian trajectory prediction, namely the Average Displacement Error (ADE), Center Average Displacement Error (CADE), and Center Final Displacement Error (CFDE), all measured in pixels.

ADE quantifies the L2-norm distance between the predicted and ground-truth bounding boxes over the entire prediction time sequence, which is defined as follows:

(27) $$ADE=\frac{1}{TS}\sum_{t=1}^{TS}{\left\|\hat{b}box_{t}-bbox_{t}\right\|}_{2}$$

where TS denotes the number of predicted time steps, $\hat{b}box_{t}$ and $bbox_{t}$ represents the predicted bounding box and the ground-truth bounding box at the time step t, respectively.

CADE quantifies the L2-norm distance between the center points of the predicted and ground-truth bounding boxes, which is defined as follows:

(28) $$CADE=\frac{1}{TS}\sum_{t=1}^{TS}{\left\|{\hat{p}}_{t}-p_{t}\right\|}_{2}$$

where TS denotes the predicted time step, ${\hat{p}}_{t}$ and $p_{t}$ denotes the center point of the predicted bounding box and the ground truth bounding box at the time step t, respectively. CFDE quantifies the L2-norm distance between the center points of the predicted bounding box and that of the ground truth bounding box at the final time step, which is defined as follows:

(29) $$CFDE={\left\|c\hat{e}n-cen\right\|}_{2}$$

where $c\hat{e}n$ and cen indicates the center point of the predicted bounding box and the ground truth bounding box at the final time step, respectively.

At present, only a limited number of public methods report pedestrian trajectory prediction results on both KITTI and SHIFT. To ensure consistency in datasets and evaluation metrics, this study mainly selects the following methods for comparison, analyzes their performance, and verifies the effectiveness of CPCAE.

PIEtraj [34] is an encoder/decoder-based model that jointly considers pedestrian intent and vehicle velocity. It leverages temporal attention and employs LSTMs to predict pedestrian trajectories. BiTraP [35] is a multi-modal model that utilizes CVAEs and RNNs to predict endpoints. A bi-directional decoder is used to improve long-term prediction accuracy. CamLiD-BiTra [36] is a multi-modal framework integrating two complementary prediction branches. The first branch captures pedestrian intent using the BiTraP architecture, while the second one simulates pedestrian motion from LiDAR data. A learnable uncertainty estimation module quantifies the reliability of each branch.

**Figure 6:**
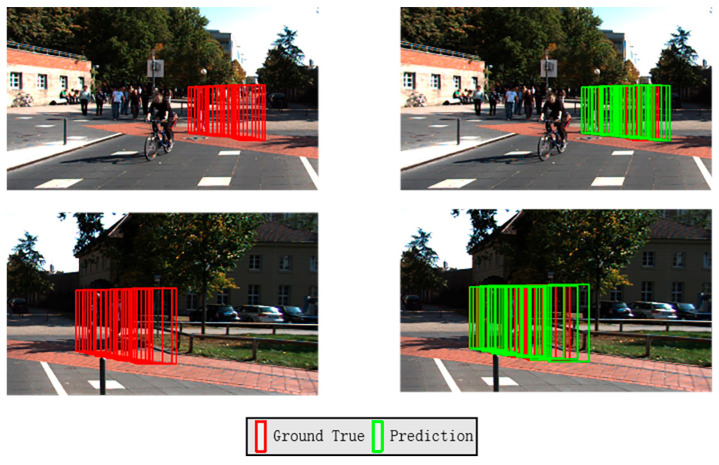
Visualization of ground-truth and predicted trajectories in a regular KITTI real-world road scene with sufficient illumination and relatively clear target contours.

Figure 6 illustrates both the ground-truth and predicted trajectories produced by the proposed method lasting for 2 s on the KITTI dataset. The left column displays the ground-truth trajectory, whereas the right column overlays our prediction with the ground truth for direct visual comparison. The results clearly demonstrate the strong predictive capability of the proposed method.

**Figure 7:**
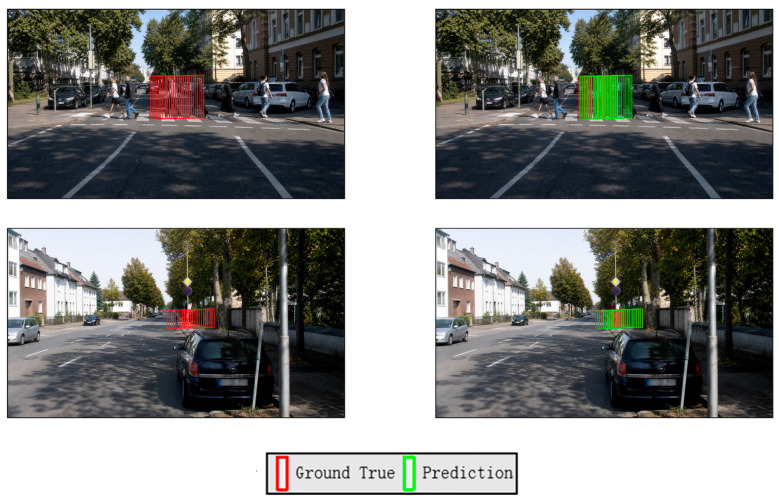
Visualization of ground-truth and predicted trajectories in KITTI real-world road scenes involving relatively crowded pedestrians and distant targets with partial occlusion.

Figure 7 presents trajectory prediction results in a relatively crowded pedestrian scene and in a distant, partially occluded scene. In the upper-left panel, corresponding to the relatively crowded scene, the predicted bounding boxes remain closely aligned with the ground-truth bounding boxes at most prediction steps, with only slight positional offsets appearing in the mid-to-late prediction horizon. In the lower-left panel, corresponding to the distant scene with partial occlusion, the predicted trajectory still follows an overall motion direction similar to that of the ground-truth trajectory. However, as the prediction horizon increases, the discrepancy between the predicted and ground-truth bounding boxes gradually becomes larger, with mild positional and scale errors. These results suggest that CPCAE can still capture the dominant motion trends of pedestrians under these two challenging scenarios, although its fine-grained long-horizon localization performance is affected by scene crowdedness, target distance, and occlusion conditions.

Table 1 evaluates the proposed CPCAE method on the KITTI and SHIFT datasets, focusing mainly on the downstream trajectory prediction performance when using compressed point cloud representations. Overall, CPCAE achieves competitive performance.

In Table 1, PIEtraj and BiTraP are selected as reference baselines. These two methods mainly rely on pedestrian trajectory information for prediction and have been evaluated on the KITTI and SHIFT datasets. The results show that CPCAE outperforms these two single-modal baselines on most evaluation metrics, indicating that the proposed multimodal architecture can effectively exploit camera-LiDAR information and improve trajectory prediction performance. CamLiD-BiTra is an existing camera-LiDAR multimodal trajectory prediction framework and is therefore selected as a strong multimodal baseline for comparison.

On the KITTI dataset, the compared methods exhibit only marginal differences in short-term prediction, indicating that pedestrian motion over a very short horizon is largely governed by historical velocity and local motion inertia. As the prediction horizon increases, CPCAE demonstrates a clearer advantage, achieving the best performance on medium- and long-horizon ADE as well as on CADE and CFDE. This suggests that CPCAE is more effective at controlling long-horizon prediction errors, particularly accumulated and final displacement errors.

On the SHIFT dataset, CPCAE shows slightly higher errors than CamLiD-BiTra. Nevertheless, its overall performance remains close to the best-performing method. This may be attributed to the larger scene scale and longer temporal span of SHIFT, where the interval between consecutive frames is relatively large. Consequently, pedestrians may undergo more substantial changes in position and motion state across adjacent frames, which increases the uncertainty of trajectory modeling. Therefore, compared with short- or medium-term prediction tasks, SHIFT places higher demands on long-term temporal modeling and fine-grained environmental perception. These findings suggest that the proposed method may be better suited to trajectory prediction scenarios with relatively shorter temporal horizons or more compact spatial scales.

CPCAE-UC denotes an uncompressed CPCAE variant without point cloud compression, while all other modules and experimental settings are kept unchanged. To examine whether point cloud compression affects downstream trajectory prediction, we further compare CPCAE with its uncompressed variant, CPCAE-UC. As shown in Table 1, CPCAE-UC achieves slightly lower prediction errors than CPCAE on both the KITTI and SHIFT datasets, suggesting that uncompressed point cloud inputs retain marginally richer geometric information for trajectory prediction. Nevertheless, the performance of CPCAE remains close to that of CPCAE-UC, demonstrating that CPCAE introduces only a minor accuracy degradation while effectively preserving task-relevant point cloud representations for downstream prediction.

In summary, the CPCAE method exhibits strong overall performance, particularly on smaller-scale trajectory prediction tasks.

**Figure 8:**
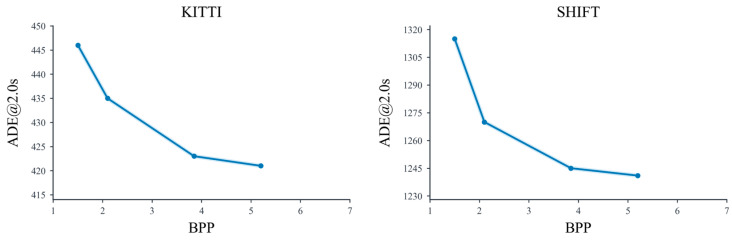
ADE@2.0s as a function of BPP on the KITTI and SHIFT datasets.

Figure 8 further illustrates the relationship between BPP and trajectory prediction accuracy. Since long-horizon prediction relies more heavily on scene geometry and pedestrian-environment interaction modeling, ADE@2.0s is selected as the primary metric for downstream prediction accuracy. On both the KITTI and SHIFT datasets, ADE@2.0s decreases as BPP increases, indicating that a higher coding rate preserves more geometric information useful for downstream trajectory prediction. At low BPP values, stronger compression leads to larger prediction errors, whereas in the high-BPP range, the error reduction gradually diminishes, suggesting that the predictive performance of the compressed representations tends to saturate. Together with the results in Table 1, these findings show that CPCAE achieves a favorable balance between point cloud compactness and downstream prediction accuracy. Therefore, the proposed method can maintain competitive trajectory prediction performance even when using compressed point cloud representations.

### 5.4. Ablation Study

To evaluate the contributions of MF-Depoco, the spatial attention module, and the modal attention module to overall performance, we conducted ablation experiments on both the KITTI and SHIFT datasets under the same experimental settings. Table 2 mainly reports the KITTI results, because the performance gains on KITTI are relatively conservative, whereas SHIFT shows consistent ablation trends with more pronounced improvements.

As shown in Table 2, either removing the spatial attention module, or replacing MF-Depoco with Depoco, or substituting the modal attention module with an MLP, all result in reduced model performance. Among these configurations, replacing MF-Depoco with Depoco causes the most significant performance degradation. These findings demonstrate that MF-Depoco, spatial attention, and modal attention each contribute positively to the overall performance, with MF-Depoco providing the most substantial improvement.

## 6. Conclusions

This paper addresses the challenges of trajectory prediction accuracy and point cloud redundancy by proposing a compressed point cloud and attention-enhanced prediction framework, termed CPCAE. The framework integrates a lossy point cloud compression scheme based on multi-feature extraction with Transformer architecture, and incorporates spatial and modal attention mechanisms to model pedestrian-environment interactions and adaptively fuse multimodal information. Experimental results further show that MF-Depoco provides effective point cloud compression, while the resulting compressed point cloud representations can support competitive downstream trajectory prediction performance in CPCAE.

Furthermore, the KITTI dataset is derived from real-world road environments and covers typical traffic scenarios such as urban roads, intersections, roadside parking, and mixed vehicle-pedestrian traffic, thereby reflecting pedestrian motion characteristics under real camera-LiDAR acquisition conditions. The SHIFT dataset further spans autonomous-driving scenarios under diverse weather, illumination, time-of-day, and scene-density conditions, including daytime and nighttime, clear and rainy weather, and sparse and relatively dense traffic. CPCAE shows competitive trajectory prediction results on both datasets, suggesting its potential to process camera-LiDAR data from different sources and under different environmental conditions, and providing preliminary evidence of its applicability to diverse road scenarios. In future real-world deployment, CPCAE may serve as a candidate multimodal modeling scheme in autonomous-driving pedestrian trajectory prediction pipelines. Given pedestrian bounding box sequences from a forward camera and road-scene point clouds from LiDAR, CPCAE could use compressed point cloud representations and attention mechanisms to fuse pedestrian motion cues with three-dimensional environmental information and generate future pedestrian position predictions. These predictions may provide useful reference information for subsequent motion planning and risk assessment.

Nevertheless, CPCAE still has several limitations. Since point cloud compression is lossy, fine-grained geometric information may be weakened in scenarios involving distant pedestrians, sparse point clouds, or severe occlusion. In addition, although the RV representation improves processing efficiency, the projection process may discard part of the original 3D spatial relationships. Future work will further optimize the trade-off between compression rate and geometric fidelity and explore lightweight network designs, semantic scene information, and cross-dataset adaptation strategies to improve robustness and deployment efficiency in real-world traffic scenarios.

## Figures and Tables

**Table 1:** Metrics of different methods on KITTI and SHIFT datasets.

Methods	KITTI			SHIFT		
	ADE	CADE	CFDE	ADE	CADE	CFDE
PIEtraj	64/131/678	535	2275	705/1126/1994	1583	5331
BiTraP	68/127/533	411	1888	745/1192/2303	2243	7473
CamLiD-BiTra	65/130/464	354	1546	386/747/1163	1123	3458
CPCAE	67/121/423	316	1470	395/770/1245	1284	3580
CPCAE-UC	65/119/418	312	1452	392/763/1236	1274	3561

Note: Table 1 reports the ADE, CADE, and CFDE metrics of different methods on the KITTI and SHIFT datasets. In the ADE columns, the three values separated by slashes correspond to prediction horizons of 0.5, 1.0, and 2.0 s, respectively, whereas CADE and CFDE are reported at the 2.0 s prediction horizon. Lower values indicate lower prediction errors.

**Table 2:** Ablation study.

Model	KITTI		
	ADE	CADE	CFDE
CPCAE	67/121/423	316	1470
-MF-Depoco + Depoco	71/129/449 ↑6.2%	337 ↑6.5%	1581 ↑7.6%
-Spatial Attention	70/127/444 ↑5.0%	333 ↑5.3%	1564 ↑6.4%
-Modal Attention + MLP	68/124/432 ↑2.2%	327 ↑3.5%	1545 ↑5.1%

Note: Table 2 reports the ablation results on the KITTI dataset using the ADE, CADE, and CFDE metrics. In the ADE column, the three slash-separated values correspond to prediction horizons of 0.5, 1.0, and 2.0 s, respectively, whereas CADE and CFDE are reported at the 2.0 s prediction horizon. The symbol “-“ denotes the removal of the corresponding module, whereas “+” denotes the replacement or addition of the following module. The symbol ↑ denotes the relative increase in error compared with the full CPCAE model; larger increases indicate more severe performance degradation.

## Data Availability

This study used and analyzed publicly available third-party datasets, including SemanticKITTI, ShapeNet, KITTI, and SHIFT. The SemanticKITTI dataset is available from its official website: https://semantic-kitti.org/dataset.html (accessed on 30 May 2026). Since SemanticKITTI is built upon the KITTI Odometry Benchmark, the corresponding Velodyne LiDAR point cloud data and calibration data should be obtained from the KITTI Vision Benchmark Suite: https://www.cvlibs.net/datasets/kitti/ (accessed on 30 May 2026), subject to the KITTI data license, registration/login procedures, and access requirements. The ShapeNet dataset is available from the official ShapeNet website: https://shapenet.org/ (accessed on 30 May 2026); access to this dataset generally requires account registration, account approval, and acceptance of the ShapeNet Terms of Use: https://shapenet.org/terms (accessed on 30 May 2026). The KITTI data used for the pedestrian trajectory prediction experiments are also available from the KITTI Vision Benchmark Suite: https://www.cvlibs.net/datasets/kitti/ (accessed on 30 May 2026), and users are required to complete the official registration/login procedures and comply with the relevant license terms. The SHIFT dataset is available from DevKit: https://www.vis.xyz/shift/ (accessed on 30 May 2026) and https://github.com/SysCV/shift-dev (accessed on 30 May 2026), and can be used for research purposes. No new dataset was created in this study.

## Abbreviations

The following abbreviations are used in this manuscript:

CPCAE	Compressed Point Cloud-based and Attention-Enhanced Trajectory Prediction Method
PCC	Point Cloud Compression
MF-Depoco	Multi-Feature Depoco
RV	Range View
BPP	Bits Per Point
SCD	Symmetric Point-to-Point Chamfer Distance
PSNR	Peak Signal-to-Noise Ratio
ADE	Average Displacement Error
CADE	Center Average Displacement Error
CFDE	Center Final Displacement Error
FP16	16-bit Floating Point
RNN	Recurrent Neural Network
LSTM	Long Short-Term Memory
CNN	Convolutional Neural Network
MLP	Multi-Layer Perceptron
FFN	Feed-Forward Network
LiDAR	Light Detection and Ranging
